# Partners matter: The psychosocial well-being of couples when dealing with endometriosis

**DOI:** 10.1186/s12955-022-01991-1

**Published:** 2022-05-28

**Authors:** Schick Maren, Germeyer Ariane, Böttcher Bettina, Hecht Stephanie, Geiser Magdalena, Rösner Sabine, Eckstein Monika, Vomstein Kilian, Toth Bettina, Strowitzki Thomas, Wischmann Tewes, Ditzen Beate

**Affiliations:** 1 Institute of Medical Psychology, Heidelberg University Hospital, Ruprecht-Karls University, Heidelberg, Bergheimer Str. 20, 69115 Heidelberg, Germany; 2 Department of Gynecological Endocrinology and Fertility Disorders, University Women’s Hospital Heidelberg, Im Neuenheimer Feld 440, 69120 Heidelberg, Germany; 3 Department of Gynecological Endocrinology and Reproductive Medicine, Medical University of Innsbruck, Anichstraße 35, 6020 Innsbruck, Austria

**Keywords:** Endometriosis, Partnership, Psychological distress, Sexuality, Social support, Actor-partner-interdependence model

## Abstract

**Background:**

Endometriosis is often associated with severe dysmenorrhea, pelvic pain and dyspareunia and has a high impact on daily life as well as sexuality. Quality of partnership positively influences the course of various diseases and ability to cope with emotional and physical distress. However, studies focusing on the male partners of endometriosis patients are rare, and even less is known about the reciprocal relationship in these couples. Therefore, this study aims to explore the interrelations in couples with endometriosis in matters of psychological distress, sexual and partnership satisfaction and social support.

**Methods:**

The cross-sectional study was conducted in two university-affiliated fertility centres in Germany and Austria with n = 104 female/male couples affected by endometriosis. Participants completed a questionnaire regarding endometriosis, partnership, sexuality, stress, anxiety, depression and social support. Both women and men were asked about the impact of women’s endometriosis-related pain (IEP) on their everyday life (e.g. leisure time). Data were analysed using the Actor-Partner-Interdependence Model.

**Results:**

Significant partner effects were evident: High depression, anxiety and stress scores in women were associated with a higher IEP in men (all *p* ≤ 0.01), reciprocally high stress and depression scores in men were correlated with a higher IEP in women (all *p* ≤ 0.05). Less sexual satisfaction in women was associated with a higher IEP in men (*p* = 0.040). There was a significant reciprocal association between the perceived lack of understanding from the social environment and a higher IEP, for both women (*p* = 0.022) and men (*p* = 0.027).

**Conclusions:**

The male partner should be taken into account when counselling or treating women with endometriosis. Our study shows a high interdependence and reciprocal influence from both partners—positively and negatively—concerning psychological distress and sexual satisfaction. Furthermore, there ought to be more awareness for the psychosocial impact of endometriosis, especially in regard to social support and understanding. Talking about and improving sexual satisfaction as well as enhancing stress reducing techniques may hold great benefits for dealing with endometriosis.

*Registration number* The study is registered with the German Clinical Trials Register (DRKS), number DRKS00014362.

## Background

Endometriosis is defined by the presence of endometrial-like tissue outside the uterus [1]. Endometriosis reacts depending on the menstrual cycle and can be asymptomatic, but is often associated with dysmenorrhea, dyspareunia and infertility. The prevalence of endometriosis is estimated to be about 5–10% in western countries [2]. According to the German IVF registry [DIR, 3], endometriosis is diagnosed in 18.5% of women with infertility, thus there is an overlap of women suffering from endometriosis and infertility. Laparoscopy remains the diagnostic gold standard and surgical treatments seem to be the most successful treatment options for endometriosis-associated symptoms [4].

There are numerous studies stating a reduced health related quality of life and psychological distress, including depression, anxiety and stress, in women with endometriosis [e.g. 5–7]. Although endometriosis is a women’s disease, the partner and the environment are important for dealing with and adjusting to the disease, since endometriosis has a high impact on patients’ daily life. However, there are only few studies about the implication of endometriosis on couples or men. For men, there are two qualitative studies dealing with the male perspective of endometriosis [8, 9]. The studies report that endometriosis has a strong impact on men’s emotions, including worry, low mood, anxiety and a grief-like process much like that experienced by their female partners, and emphasize the lack of support available to men.

Sexuality is an important aspect when dealing with endometriosis, since dyspareunia is one of the key symptoms. Accordingly, there are many studies reporting impaired sexuality in women suffering from endometriosis [10–12] and subsequent important implications for quality of life in women [13, 14]. Regarding the male perspective, there are two quantitative studies comparing sexual functioning of male partners of endometriosis patients to control couples [15, 16], with diverging results regarding the increase of sexual dissatisfaction in male partners.

A growing body of research emphasizes the major psychological effects of chronic illnesses on partners [e.g. 17–21]. Hudson et al. [22] highlight the opportunity of dyadic analysis in capturing the social and relational implications of health and illness on social networks—and its consequences of being a source of support or additional stress. There are three qualitative studies assessing coping processes of endometriosis in a couple setting [23–25]. These studies report an immense strain of endometriosis on relationships, attributed to social withdrawal, dyspareunia and difficulties of partners to tolerate the constancy of the disease. To our knowledge, there is just one quantitative study exploring the distinct associations of emotional intimacy, empathic concern and relationship satisfaction in couples living with endometriosis, emphasizing the interrelations between patients and their partners [26].

In sum, quantitative studies focusing on endometriosis and the specific reciprocal relationships in couples are missing. Therefore, the ESHRE Guideline on management of women with endometriosis [1], Culley et al. [27] and Quinlivan et al. [28] emphasize the need for more research focusing on the psychosocial impact of endometriosis on women and partners.

Thus, the main research objective of our exploratory study is to resolve the question, how partners of romantic couples reciprocally influence each other in dealing with the impact of endometriosis. Based on the previous (scarce) literature on the psychological impact of endometriosis on women, men and couples, we hypothesize that each partner’s (1) psychological distress, (2) sexual satisfaction, (3) partnership satisfaction and (4) social support influence the other partner’s dealing with the impact of endometriosis.

To test our hypotheses, we perform dyadic data analysis to evaluate the effects psychological distress, sexual satisfaction, partnership and social support can have on women and men towards the impact of endometriosis.

## Methods

### Setting

A quantitative cross-sectional multi-centre study was conducted at the University Hospital Heidelberg (UKHD), Germany and the Medical University of Innsbruck (MUI), Austria. The study was approved by the Ethics Committee of the Heidelberg Medical Faculty (S-301/2016) and Medical University of Innsbruck (AN-2017-0028 370/4.7). All women undergoing diagnostic laparoscopy to explore the reason for dysmenorrhea or infertility during their treatment at UKHD and MUI and their partners were invited to participate in the study.

At UKHD data was collected at the Department of Gynaecological Endocrinology and Fertility Disorders from September 2016 to August 2018. All patients and their partners were informed about the study during a pre-operation consultation before the laparoscopy. Patients also had the opportunity to take home the documents for their (not accompanying) partners or to participate alone. Participants who agreed to take part were asked to fill in the questionnaires in a timely manner after the laparoscopy. All Participants were informed about their right to withdraw from participation consent at any time without giving any reasons and without any consequences. At MUI the survey took place at the Department of Gynaecological Endocrinology and Reproductive Medicine from June 2017 to July 2018. Patients and their partners were recruited in the same manner as at the UKHD.

### Sample

In total, about 800 questionnaires were handed out. N = 322 individuals took part in the study. For answering the main research objective in this paper, we included only women with clinically proven endometriosis whose partners were also participating in the study. Thus, our sample consisted of n = 104 couples.

Mean age of women was 33.0 years (SD 5.47), for men 36.2 years (SD 6.42). Of all participants, 59.4% were high school or university graduates. Mean partnership duration was 8.93 years (SD 5.23). Due to the clinical confirmation per laparoscopy, surgical treatment was the most mentioned treatment by women (86.7%), followed by hormonal treatment (41%), naturopathic treatment (15.8%) and other pain therapy (9.6%). Of all participants, 31.4% already had children, 74.0% had a present wish for a child, on average for 3.93 years (SD 3.08), and reported infertility treatment on average for 2.01 years (SD 2.26).

### Questionnaires

#### Clinical and sociodemographic data

Clinical data on the laparoscopy results were retrieved from the patients’ medical records. Psychometric parameters were assessed using questionnaires as part of self-evaluation instruments.

The questionnaire contained socio-demographic information regarding age, education, occupation, family status, partnership duration, endometriosis (duration, treatment) and wish for a child (reason for infertily, duration, treatment). The selection of questionnaires and psychosocial variables was based on the current state of studies and clinical experience from a variety of consultations and counselling sessions. In total, the questionnaire for women had up to 88 items and for men up to 72 items, depending on the presence of endometriosis and wish for a child. In the following, only the questionnaires used for this paper’s analyses are reported.

#### Impact of endometriosis-related pain (IEP)

The impact of endometriosis-related pain (IEP) on everyday life for women and men, our dependent variable, was assessed with visual analogue scales (VAS) from the German pain questionnaire [DSF; 29]. For women, we asked, e.g. “To what extent has the pain in the last 3 months affected your recreational activities or family or circle of friends?”. Items for men were, e.g. “To what extent has your partner’s pain in the last 3 months affected your leisure activities or activities with family or friends in the last 3 months?”. Furthermore, we asked women about their pain intensity, e.g. “Indicate the intensity of your current pain/ average pain over the last 4 weeks”. Participants had to rate the items from 0 (“not at all”) to 10 (“very much”).

#### Information about endometriosis status

Participants’ information about endometriosis status was assessed with the short version of the Endometriosis Health Profile (EHP-5) on a five-point Likert Scale with the answer options from 1 (“never”) to 5 (“always”) and with Cronbach’s alpha ranging from 0.79 to 0.97 [30]. We adjusted the 11 EHP-5 items for the male partner, e.g. “I felt frustrated because treatment is not working” to “I was frustrated because the treatment didn’t work for my partner".

We focused specifically on the EHP social support item “I felt others do not understand what I’m going through” (for women) or “… what my partner is going through” (for men) to capture the social impact of endometriosis on women’s and men’s life.

#### Psychological distress

The German version of the Depression, Anxiety and Stress Scales [DASS; 31] was used to measure psychological distress, including depression (e.g., “I felt that I had nothing to look forward to”), anxiety (e.g., “I experience trembling”) and stress (e.g., “I found it hard to wind down”). The DASS is a 21-item scale with all items rated on a four-point Likert Scale from 0 (never) to 3 (almost always). Cronbach’s alpha of the DASS for the depression subscale is 0.91, for the anxiety subscale 0.78–0.82 and for the stress subscale 0.81–0.89.

#### Sexuality

Sexual satisfaction was assessed on a VAS from 0 (“not satisfied”) to 10 (“very satisfied”). Sexual intercourse frequency and (female) pain during sexual intercourse were assessed on a five-point Scale with the answer options “no”, “once/month”, “once/week”, “several/week” and “no statement”. The items were partly derived from the Female Sexual Function Index [FSFI; 32].

#### Partnership and social support

For the evaluation of partnership satisfaction and social support we used 6 items from the Swiss Household Panel [SHP; 33] on a VAS from 0 (“not at all”) to 10 (“very much”). We asked to evaluate “To what extent can your partner / your relatives, friends, acquaintances or work colleagues provide you with practical help” and “To what extent is your partner/are your relatives, friends, acquaintances or work colleagues available in case of need and show understanding?” Furthermore, for partnership satisfaction we asked to rate the intimacy and happiness of their partnership.

### Statistical analysis

Assuming a medium effect size (ES) of d = 0.30, a total of 79 dyads need to be recruited to have adequate power to detect actor and partner effects at an alpha level of α = 0.05. Statistical analysis was performed using the Statistical Package for Social Sciences (SPSS) for Windows, Version 25 (IBM Corp. 2017). Data were analysed with a couple as the unit of analysis. The clinical and socio-demographic variables were described through descriptive statistics. Comparisons of means between men and women were run by paired *t*-tests. For dyadic data analysis, the Actor Partner Interdependence Model [APIM; 34] was used. The APIM takes couples as the unit of analysis and stipulates that one person’s behaviour not only affects him-/herself (actor effect) but also his/her partner (partner effect) [35].

In all our APIM analyses, impact of endometriosis-related pain was the dependent variable and depression, anxiety, stress, sexual satisfaction, partnership and social support were each individual independent variables, calculated separately for women and men. To calculate the APIM, we used SPSS Syntax Mixed and the web-based program APIM_MM (Actor Partner Interdependence Model with Multilevel Modelling) for distinguishable dyads by David A. Kenny (http://davidakenny.net/DyadR/DyadRweb.htm). This program also calculates ES to estimate the clinical relevance of the APIM effects. *p* < 0.05 was considered statistically significant. The assumption of normal distribution was violated for the variables ‘impact of endometriosis-related pain’, ‘partnership’, ‘sexual satisfaction’, ‘depression’, and ‘anxiety’. We performed square root transformation to achieve a normal distribution for these variables. Subsequently all predictors were centred [36].

## Results

Paired *t*-test analysis showed major differences in numerous self-report outcomes between women and men (Table 1). Women had significant higher values concerning depression, anxiety and stress (all *p* ≤ 0.001), social support (*p* = 0.010) and IEP (*p* < 0.001).

**Table 1:** Sociodemographics

Variables	Women mean (SD) or n (%)	Men mean (SD) or n (%)	*p*-value (paired *t*-test)
**Sociodemographics**			
Age	33.02 (5.47)	36.17 (6.42)	0.000
Partnership in years	8.98 (5.18)	8.88 (5.31)	0.478
Existing own children	31 (29.80)	34 (33.00)	
*Education*			
No graduation	–	1 (1.00)	
Secondary school	41 (39.90)	42 (40.90)	
Abitur	21 (20.40)	18 (17.50)	
University	41 (39.90)	42 (40.90)	
**Endometriosis**			
Duration of treatment in years	2.51 (3.65)	–	
*Impact of endometriosis pain on everday*			
life activities	32.43 (34.00)	21.36 (28.44)	0.000
**Wish for a child**			
Wish for a child in years	3.92 (2.83)	3.91 (3.38)	1.000
Duration of infertility treatment in years	2.09 (2.22)	1.91 (2.30)	0.208
**Psychosocial Well-Being**			
Depression	4.21 (4.89)	1.86 (2.51)	0.000
Anxiety	3.19 (3.86)	1.03 (1.73)	0.000
Stress	6.93 (4.73)	3.72 (3.43)	0.000
Social Support	7.03 (2.48)	6.31 (2.51)	0.010
Partnership satisfaction	8.91 (1.07)	9.07 (1.26)	0.262
Sexual satisfaction	7.08 (3.03)	7.37 (2.60)	0.984

Stage of endometriosis according to the revised American Society for Reproductive Medicine [37] was assessed in n = 98 women (94.2%), and showed no significant correlations to pain intensity (r =  − 0.051, *p* = 0.637) or stress score (r =  − 0.003, *p* = 0.979).

### Impact of endometriosis-related pain

All APIM couple analyses (see Table 2) were controlled for partner’ age and wish for a child, where necessary, APIM couple analysis were additional controlled for sex frequency and social support.

**Table 2:** Actor–partner interdependence model with impact of endometriosis pain as the dependent variable and all predictors

	Impact of endometriosis pain		
	ß	t	*p*
**Model 1: Depression (n = 97 couples)**			
*Actor effects*			
Women	0.216	2.107	0.036
Men	0.206	1.940	0.054
*Partner effects*			
Women	0.317	2.514	0.013
Men	0.243	2.769	0.006
**Model 2: Anxiety (n = 97 couples)**			
*Actor effects*			
Women	0.389*	4.268	0.001
Men	0.156	1.442	0.151
*Partner effects*			
Women	0.250	1.960	0.052
Men	0.316*	4.000	0.001
**Model 3: Stress (n = 97 couples)**			
*Actor effects*			
Women	0.283	2.422	0.016
Men	0.160	1.751	0.082
*Partner effects*			
Women	0.276	2.574	0.011
Men	0.230	2.112	0.036
**Model 4: Sexual satisfaction (n = 84 couples)**			
*Actor effects*			
Women	− 0.225	1.660	0.099
Men	− 0.098	0.756	0.451
*Partner effects*			
Women	− 0.038	0.260	0.795
Men	− 0.244	2.068	0.040
**Model 5: EHP Support (n = 67 couples)**			
*Actor effects*			
Women	0.421*	3.212	0.002
Men	0.364*	2.795	0.006
*Partner effects*			
Women	0.342	2.324	0.022
Men	0.255	2.244	0.027

Covariates: Model 1–5: age, wish for a child; Model 4: sex frequency; Model 5: social support

*displaying a medium effect size

#### Psychological distress

Women who indicated higher depression scores showed a higher IEP (*p* = 0.036), and also their partners reported a higher IEP (*p* = 0.006). Thus, women’s depression were significantly linked to how men evaluated the impact of endometriosis on their own everyday life. Furthermore, there was also a women partner effect: men’s depression scores were associated with women’s IEP (*p* = 0.013).

For anxiety, women showed a significant medium actor effect on IEP (*p* = 0.001). Moreover, women’s anxiety scores were significantly associated with how the men evaluated the IEP on their everyday life (*p* = 0.001).

**Fig. 1:**
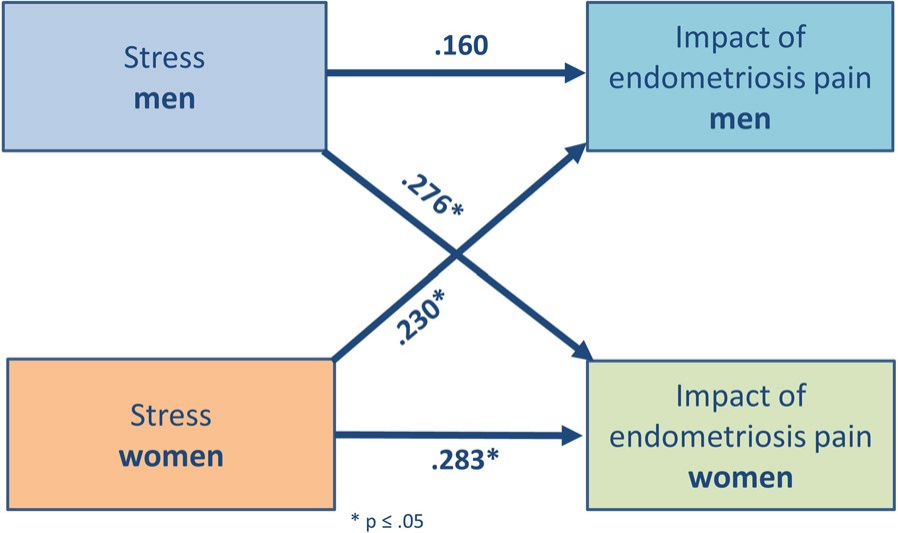
Actor partner interdependence model for effect of stress on impact on endometriosis-related pain (IEP). Women’s stress scores are associated with a higher IEP in women (actor effect) and men (partner effect). Men’s stress scores correlates positively with women’s IEP (partner effect). * all *p* < 0.05

Women who reported greater stress indicated a higher IEP (*p* = 0.016). Furthermore, we found significant partner effects: Women’s stress scores were associated with men’s IEP (*p* = 0.036) and men’s stress scores with women’s IEP (*p* = 0.011) (see Fig. 1).

#### Sexual satisfaction

**Fig. 2:**
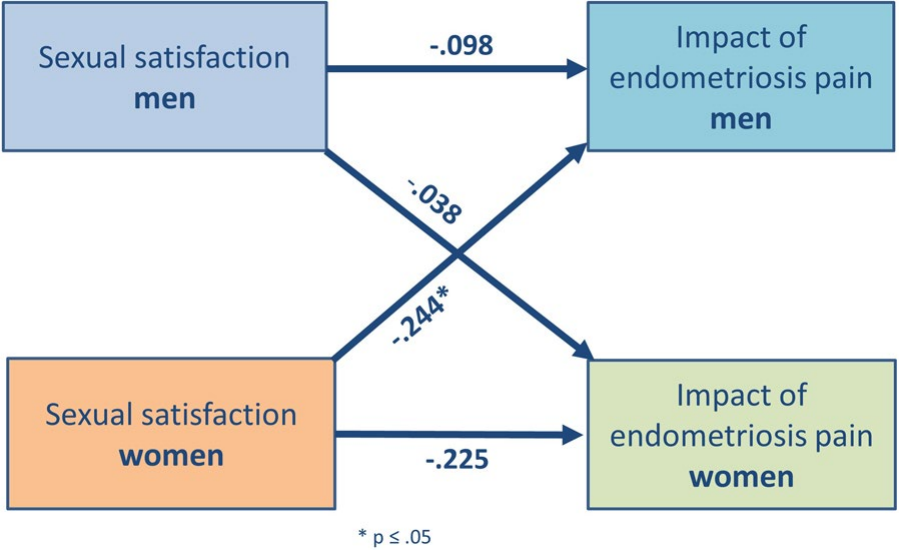
Actor partner interdependence model for effect of sexual satisfaction on IEP. Women’s sexual satisfaction correlates negatively with men’s IEP (partner effect). The rating of men’s sexual satisfaction does not influence women’s IEP. * *p* < 0.05

There was a significant negative partner effect concerning women’s sexual satisfaction and the IEP in men (*p* = 0.040). Thus, the lower women rated their sexual satisfaction, the higher men reported an impact of endometriosis on their lives (see Fig. 2).

#### Partnership and social support

Partnership satisfaction and social support (assessed with the SHP) had no influence on the IEP for women and men. Thus, we found neither actor nor partner effects of partnership satisfaction or social support towards women’s or men’s IEP.

**Fig. 3:**
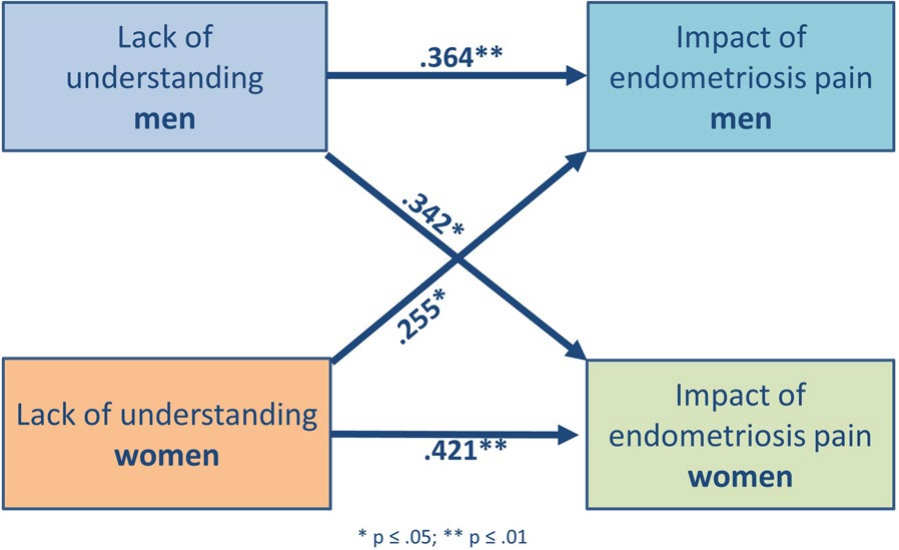
Actor partner interdependence model for effect of lack of understanding from others on IEP. Women’s reported lack of understanding from others is associated with a higher IEP in women (actor effect) and men (partner effect). Men’s reported lack of understanding from others is associated with a higher IEP in men (actor effect) and women (partner effect). * all actor effects *p* < 0.01, all partner effects *p* < 0.05

However, regarding the comprehension of endometriosis from the social environment, there seems to be an apparent lack of understanding towards the impact of endometriosis-related pain on the lives of women and men (see Fig. 3). We found significant actor effects for women (*p* = 0.002) and men (*p* = 0.006) on the EHP social support item “perceived lack of understanding”, displaying a medium ES. Furthermore, we found two significant partner effects from women’s evaluation of social understanding to men’s IEP (*p* = 0.027) and from men’s evaluation of social understanding to women’s IEP (*p* = 0.013). Thus, the more men and women thought that others would not understand what endometriosis meant, the higher they reported a negative impact of endometriosis-related pain on their everyday life. These effects were stable when controlling for the social support items of the SHP.

## Discussion

In the present study, we address the significant gap in research concerning the relations in couples with endometriosis in matters of psychological distress, sexuality, partnership satisfaction and social support. Our hypotheses that each partner’s (1) psychological distress, (2) sexual satisfaction, (3) partnership satisfaction and (4) social support influence the other partner’s dealing with the impact of endometriosis, have partly been confirmed. Thus, the key findings of this study may be summarized as follow: (1) Psychological distress is related to actor and partner effects on IEP within the dyad. (2) A significant interaction was observed between women’s lower sexual satisfaction and men’s higher IEP. (3) Partnership satisfaction had no impact on how women and men influence each other while dealing with endometriosis. (4) For women and men, we found a significant relation concerning the perceived lack of understanding from the social environment and a higher IEP.

Our results indicate that couples are highly interdependent in their evaluation of and emotional response to endometriosis, and show reciprocal influences on how their psychological distress, sexual satisfaction, and perceived lack of understanding from others are associated to the impact of endometriosis-related pain on their everyday life.

### Psychological distress

The adverse relationship between endometriosis and psychosocial well-being in women has been shown in various studies and reviews [16, 27, 38–40]. In a cross-sectional study including over 10,000 women an increased risk of developing major depression and anxiety disorders among women with endometriosis was present [41]. The highly significant results for depression, anxiety and stress in our study demonstrate the close relationship with the impact of endometriosis-related pain in women and demand empathic consultations and awareness from health professionals for the psychosocial risks of endometriosis. For male partners of women with endometriosis, qualitative studies point out that endometriosis can pose a challenge to their own quality of life [8, 9]. Those findings are in line with our study results, which indicate that also men are affected by the impact of endometriosis on their (social) lives. Mittinty et al. [21] analysed dyadic coping behaviour in patients with chronic pain and their spouses and found reciprocal relationships with depression, anxiety and stress. Thus, there seems to be a high interconnectedness within couples with chronic diseases in regard to psychological distress. The reciprocal effects of endometriosis on couples’ lives regarding psychosocial distress shown in our study is an additional example of the so called “We-disease” [42], defined as shared appraisal and shared efforts to cope with a disease in couples [43], which needs to be addressed by psychosocial care in clinics.

### Sexuality

Sexuality is a relevant issue when dealing with endometriosis. We found a negative relationship between sexual satisfaction in women’s and men’s reported IEP on their daily lives, so less sexual satisfaction in women was associated with a higher impact of endometriosis pain in men. Previous research in women with endometriosis highlighted the adverse effects of endometriosis on sexual functioning and sexual quality of life [10, 11, 13, 14], especially the negative implications of dyspareunia on partner intimacy and relational distress [44]. Indeed, significant partnership problems caused by endometriosis-related symptoms and even break-ups or divorces have been reported [5, 45]. Thus, there seems to be a particular need to include the partner’s perspective when coping with endometriosis [46]. Compared with the two quantitative studies regarding male sexuality and endometriosis [15, 16], our study supports the results of sexual implications for male partners of endometriosis patients [15]. However, we found no direct actor effect in men concerning their sexual satisfaction and the impact of endometriosis on their lives, which may be due to a male social desirability in reporting sexual satisfaction. To our knowledge, these are the only quantitative studies regarding this topic, thus more research is needed, especially with focus on couple analysis.

### Partnership

In our study, we found no direct effects of partnership satisfaction on the impact of endometriosis. This may be due to the nearly equally very high partnership satisfaction in our sample, with women and men rating their satisfaction on average with 8.91 (SD 1.07) respective 9.07 (SD 1.26) on a scale from 0 to 10. Nevertheless, focusing on a good partnership is important, since relationship satisfaction has positive effects on the management of different kinds of mental and physical diseases with improved recovery [47]. On the other hand, Pluchino et al. [46] point out that relationship problems in endometriosis couples were attributed to decreased socialization due to the chronic disease and the incapacity of partners to tolerate the endometriosis symptoms. Considering the vicious and the supportive circle, it is important to take the couple and their relationship into account, when diagnosing, treating and supporting women with endometriosis.

### Social support vs. lack of understanding

Social interactions have a profound impact on our ability to cope with emotional and physical distress [48]. The significant relationship in our study between lack of understanding and higher IEP was the most momentous one. Particularly since the regular social support items from the SHP—regarding the availability of relatives, friends, acquaintances and work colleagues being there if necessary—had no impact on IEP, neither as a predictor nor as a covariate variable. Thus, participants seem to differentiate between the feeling of having good social support vs. the feeling others could understand what having endometriosis meant for their lives or the lives of their female partners.

Qualitative research has highlighted the strain of endometriosis symptoms on social relationships and consequently social support [24, 49]. For male partners, Culley et al. [8] indicated the lack of support available to men and the absence of professional or societal recognition of the impact on male partners of endometriosis patients. Whitney [50] reported that women wanted others to understand and to share information about the symptoms of endometriosis. The quality of one’s social relationships is reliably related to positive and negative physical health outcomes [51, 52]. Therefore, a social surrounding that is less understanding can have adverse effects on health—on an individual and reciprocal level in endometriosis patients and their partners. Thus, support groups for endometriosis are highly recommended. Given that only 20% of the population seem to know about endometriosis [53] and the effects endometriosis can have on a woman’s life and partnership, our data mirror what patients and their partner are experiencing. There are different reasons why endometriosis—despite its prevalence—is rarely known in society. One important reason is the various, complex appearance of endometriosis, from asymptomatic to monthly dysmenorrhea to heavy pain and infertility. Another reason is that dysmenorrhea as a symptom of endometriosis is neglected in society as “menstrual cramps are normal” [28, 54]. Awareness for this “chameleon of gynaecology” [55] is highly needed to reduce time from symptom onset to diagnosis and to increase understanding for endometriosis in the public—for the concerned women and their partners.

### Strength and limitations

Our study has several strengths: There are only few studies investigating the attitudes and experiences of couples with women suffering from endometriosis respectively the attitudes and experiences of the male partner. Thus, our study results extend the existing literature in many psychosocial aspects. Furthermore, our study examines a broad clinical picture, displaying a high external validity. Using dyadic data analysis promotes a meaningful examination of the association between stress, anxiety, depression, sexual satisfaction and IEP. In addition, the number of participating men is high, we had n = 104 couples in our sample and only 24 individual female participants (not reported in this paper); thus, in most cases both partners participated. In addition, endometriosis was clinically confirmed by laparoscopy. Furthermore, our study was conducted at two study locations: Germany and Austria, which enriches the external validity of the results.

The study reveals the following limitations. First, the cross-sectional study design limits conclusions on causal relationships or possible changes over time in the interaction between couples. Second, for the most part, information was achieved via self-report measures. Thus, the data may suffer from reporting bias. Furthermore, the adjustment to the specific situation in endometriosis, e.g. the adapted male version of the EHP-5 as investigator-derived questions, likely influenced the validity of the questionnaires. Third, generalization of findings is limited to couples who attended treatment in a university hospital setting, as often the case in clinical studies with endometriosis patients [e.g. 5, 56]; therefore, allowing the risk of selection bias. Moreover, due to our recruiting strategy, there is a huge overlap of couples with endometriosis and wish for a child. However, our major outcome are the interrelationships of endometriosis-related pain, which are detached from the wish for a child.

Fourth, a study bias might exist since couples were addressed at the same time-point. Contrary to instructions, couples might have answered their questionnaires together or one person might have urged the other person to participate. Fifth, participants in this sample had an above-average high educational background and show a limited age range.

## Conclusions

Our study results show a high interdependence and reciprocal influence from both partners—positively and negatively—on psychological distress, sexual satisfaction and perceived understanding from others when dealing with the women’s endometriosis. This suggests that so far the impact of endometriosis on the partner has been underestimated. The present data suggests that the partner should be taken into account when counselling women on how to cope with endometriosis in daily routine. Furthermore, it is important to talk about and improve sexual satisfaction as well as to enhance stress reducing techniques for both partners, which may hold large benefits for dealing with endometriosis.

Above this, public awareness for the psychosocial impact of endometriosis, especially about the social consequences for the affected women and their partners, should be further increased.

Our findings suggest that couple-based studies and an attempt to include the partners when collaboratively manage endometriosis, can substantially improve women’s coping with this chronic disease.

## Data Availability

The datasets used and/or analysed during the current study are available from the corresponding author on reasonable request.
