# Correction: Partners matter: The psychosocial well-being of couples when dealing with endometriosis

**DOI:** 10.1186/s12955-022-02027-4

**Published:** 2022-07-28

**Authors:** Maren Schick, Ariane Germeyer, Bettina Böttcher, Stephanie Hecht, Magdalena Geiser, Sabine Rösner, Monika Eckstein, Kilian Vomstein, Bettina Toth, Thomas Strowitzki, Tewes Wischmann, Beate Ditzen

**Affiliations:** 1 Institute of Medical Psychology, Heidelberg University Hospital, Ruprecht-Karls University, Heidelberg, Bergheimer Str. 20, 69115 Heidelberg, Germany; 2 Department of Gynecological Endocrinology and Fertility Disorders, University Women’s Hospital Heidelberg, Im Neuenheimer Feld 440, 69120 Heidelberg, Germany; 3 Department of Gynecological Endocrinology and Reproductive Medicine, Medical University of Innsbruck, Anichstraße 35, 6020 Innsbruck, Austria

## Correction to: Health and Quality of Life Outcomes (2022) 20:86 10.1186/s12955-022-01991-1

The original article mistakenly inverted the names of all authors; this has since been corrected.

